# Lung Ultrasound to Evaluate Fluid Status and Optimize Early Volume-Expansion Therapy in Children with Shiga Toxin-Producing Escherichia Coli–Haemolytic Uremic Syndrome: A Pilot Study

**DOI:** 10.3390/jcm13113024

**Published:** 2024-05-21

**Authors:** Marco Allinovi, Ilaria Farella, Martina Giacalone, Gianmarco Lugli, Luigi Cirillo, Niccolò Parri, Francesca Becherucci

**Affiliations:** 1 Nephrology, Dialysis and Transplantation Unit, Careggi University Hospital, 50134 Florence, Italy; gnm.lugli@gmail.com; 2 Clinica Medica “A. Murri”, Department of Biomedical Sciences & Human Oncology, University of Bari “Aldo Moro”, 70121 Bari, Italy; ilaria.farella@uniba.it; 3 Department of Emergency Medicine and Trauma Center, Meyer University Children’s Hospital IRCCS, 50139 Florence, Italy; martina.giacalone@meyer.it (M.G.); niccolo.parri@meyer.it (N.P.); 4 Nephrology and Dialysis Unit, Meyer Children’s Hospital IRCCS, 50139 Florence, Italy; luigi.cirillo@meyer.it (L.C.); francesca.becherucci@meyer.it (F.B.); 5 Department of Biomedical, Experimental and Clinical Sciences “Mario Serio”, University of Florence, 50121 Florence, Italy

**Keywords:** Shiga toxin-producing Escherichia coli–haemolytic uremic syndrome, thrombotic microangiopathy, haemolytic–uremic syndrome, lung ultrasound, B-lines

## Abstract

**Background:** Shiga toxin-producing Escherichia coli–haemolytic uremic syndrome (STEC-HUS) can result in kidney and neurological complications. Early volume-expansion therapy has been shown to improve outcomes, but caution is required to avoid fluid overload. Lung ultrasound scanning (LUS) can be used to detect fluid overload and may be useful in monitoring hydration therapy. **Methods:** This prospective observational pilot study involved children with STEC-HUS who were recruited from a regional paediatric nephrology centre. B-line quantification by LUS was used to assess fluid status at the emergency department (ED) admission and correlated with the decrease in patient weight from the target weight. A control group of children on chronic dialysis therapy with episodes of symptomatic fluid overload was also enrolled in order to establish a B-line threshold indicative of severe lung congestion. Another cohort of “healthy” children, without renal or lung-related diseases, and without clinical signs of fluid overload was also enrolled in order to establish a B-line threshold indicative of euvolemia. **Results:** LUS assessment was performed in 10 children with STEC-HUS at ED admission, showing an average of three B-lines (range 0–10). LUS was also performed in 53 euvolemic children admitted to the ED not showing kidney and lung disease (healthy controls), showing a median value of two B-lines (range 0–7), not significantly different from children with STEC-HUS at admission (*p* = 0.92). Children with STEC-HUS with neurological involvement during the acute phase and those requiring dialysis presented a significantly lower number of B-lines at admission compared to patients with a good clinical course (*p* < 0.001). Patients with long-term renal impairment also presented a lower number of B-lines at disease onset (*p* = 0.03). **Conclusions:** LUS is a useful technique for monitoring intravenous hydration therapy in paediatric patients with STEC-HUS. A low number of B-lines at ED admission (<5 B-lines) was associated with worse short-term and long-term outcomes. Further studies are needed to determine the efficacy and safety of an LUS-guided strategy for reducing complications in children with STEC-HUS.

## 1. Introduction

Haemolytic–uremic syndrome (HUS) caused by Shiga toxin-producing Escherichia coli (STEC) infection (STEC-HUS) is the most frequent cause of acute kidney injury (AKI) in children, with a mortality rate ranging between 3 and 5% [1,2]. During the acute phase, 47–70% of patients may require kidney replacement therapies, and 25–60% of cases have long-term renal sequelae, namely hypertension, proteinuria, chronic kidney disease (CKD), and end-stage kidney disease (ESKD) [3,4,5].

The pathophysiology of AKI in STEC-HUS is multifactorial, involving microvascular kidney damage from direct endothelial toxicity of Shiga toxin, and pre-renal ischemia due to absolute or relative hypovolemia. Indeed, muco-hematic diarrhoea, vomiting, fever, and fasting are typical features of STEC-HUS, all leading to hypovolemia, especially at the onset of the disease [6,7,8,9].

Notwithstanding this, assessing the volume status of patients with STEC-HUS at onset can be difficult. Dehydration and AKI can be associated with fluid overload due to kidney injury, causing clinical overlapping of hypo- and hypervolemia signs/symptoms [due to oligoanuria, hypoalbuminemia, and third spaces (such as ascites, pleural or pericardial effusion)] [10], and challenging treatment.

In recent years, early and massive-volume expansion therapy (up to 10% of the target weight) has proven to be associated with a lower incidence of oligoanuria, a lower need for dialysis, improved medium and long-term renal outcomes, and a lower incidence of acute neurological complications in STEC-HUS [5,11,12,13]. Of note, both the two edges of volume status, hypovolemia and hypervolemia, are associated with a higher mortality in children with STEC-HUS [5,6,7,8,9,11,12,13,14]. Therefore, a precise water balance is critical for these patients, as seen in other forms of AKI, with the aim of avoiding hypovolemia on one hand and fluid overload on the other.

Several methods have been proposed for fluid status assessment, and dynamic indices should be preferred in critically ill patients [15]. The use of lung ultrasound (LUS) to assess the state of hydration in patients with AKI is receiving increasing attention for its effectiveness, ease of use, and low costs [16]. Additionally, LUS has a potential role in detecting lung congestion even at a pre-clinical stage, in patients with AKI [17].

We hypothesize that LUS may accurately evaluate fluid status at emergency department (ED) admission in children with AKI secondary to STEC-HUS, and we explore its role in optimizing volume administration and ameliorating kidney outcomes.

## 2. Materials and Methods

### 2.1. Study Population

In this prospective observational pilot study, all consecutive children with a diagnosis of STEC-HUS presenting to the ED of Meyer University Children’s Hospital IRCCS (Florence, Italy) between 1 June 2018 and 1 November 2020 were enrolled in the study. STEC-HUS was defined as the presence of microangiopathic haemolytic anaemia, thrombocytopenia, and AKI in the presence of laboratory confirmation of STEC infection. Criteria for microbiological confirmation were the following: (1) isolation of an E. coli strain producing Shiga toxin by culture, or (2) direct detection of stx1 or stx2 nucleic acid (without strain isolation) by real-time PCR, or (3) detection of E. coli serogroup-specific antibodies.

Inclusion criteria were the following: (1) confirmation of STEC diagnosis in patients with HUS; and (2) LUS and inferior vena cava (IVC) ultrasound to assess the volume status immediately at ED admission.

Exclusion criteria were the following: (1) a medical history of pneumonia in the previous weeks; (2) previous diagnosis of lung fibrosis or interstitial lung disease, which are diseases that appear as multiple B-lines during an LUS, regardless of the fluid status; (3) age at onset of STEC above 18-years-old; and (4) a confirmed genetic diagnosis of atypical HUS (aHUS) (known at the end of follow-up). We also excluded from this analysis those children with STEC-HUS who underwent eculizumab therapy, because it proved to prevent/mitigate long-term kidney sequelae with mechanisms unrelated to fluid status correction [18]. All the patients were followed for at least 6 months after diagnosis, monitoring proteinuria, renal function and blood pressure.

### 2.2. Clinical and Anthropometric Data at Tertiary Hospital Admission

At ED admission at our tertiary hospital, anamnestic data and the following clinical parameters were assessed: weight, blood pressure, fluid status, and urinary output. Blood pressure was measured manually with a sphygmomanometer and stethoscope; hypertension was defined following the National High Blood Pressure Education Program [19]. Demographic data and previous medical history were extracted from the participant’s medical records and direct interviews. At ED admission, hypovolemia and euvolemia were defined according to the weight change calculated from (1) a reference weight reported by parents or paediatrician in the last month before admission; and (2) the weight expected corresponding to the growth percentile of the last year followed by the child. Oliguria was defined as a urine output of less than 0.5 mL/kg/h in infants or less than 1 mL/kg/h in older children for a duration of at least 6 h. Patients were categorized into two groups according to the extent of hydration therapy (protocol of rehydration) performed in different hospitals before admission at our tertiary hospital: (1) patients who received a robust expansion volume (EV+), and (2) patients who received hydration therapy following standard prescription (EV−). The EV+ patients received 10 to 15 mL/kg/h infusions of normal saline from the time of diagnosis of HUS, according to the protocol described by Ardissino et al. [12]; EV− patients received hydration therapy as per the local hospital protocol, based on a clinical assessment and never exceeding 10 mL/kg/h. All the patients received volume expansion therapy initiated within 6 h of hospital admission and continued to maintain a target weight (up to 10% of working weight).

### 2.3. Laboratory Measurement at Tertiary Hospital Admission

In all patients, serum creatinine, haematocrit, urea, sodium, and albumin were recorded at the time of ED admission. All these measurements were performed in the central laboratory of Meyer Children’s Hospital IRCCS. STEC diagnosis was established by stool culture and PCR at the national reference laboratory (Istituto Superiore di Sanità, Rome, Italy).

### 2.4. Ultrasound Measurements

**Figure 1:**
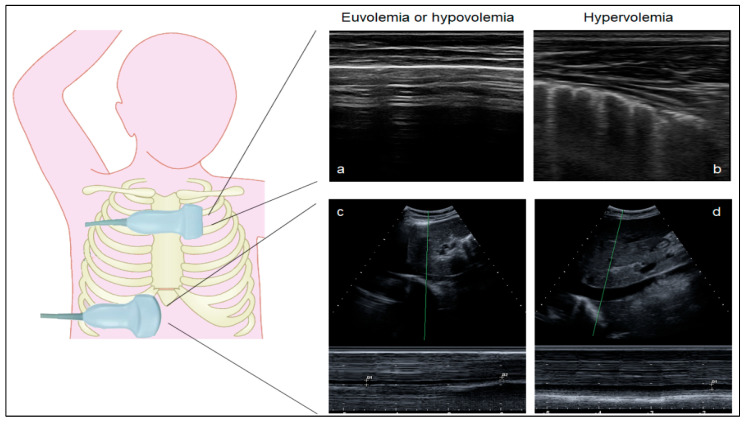
Ultrasound assessment of B-lines in different conditions of volume status. Ultrasound assessment of B-lines at lung ultrasound and inferior vena cava diameters in euvolemia or hypovolemia (**a**,**c**) and hypervolemia (**b**,**d**). (**a**) Normal lung: horizontal hyper-echogenic lines represent the lung pleura interface (A-lines). (**b**) Pulmonary congestion: hyperechogenic B-lines with a narrow base radiating from the transducer to the lower border of the window. Ultrasound of the inferior vena cava, measured on M-mode, is a sensitive method in volume status monitoring. The IVC is explored in the subxiphoid window in its sagittal view, measuring the maximum IVC diameter (IVCmax) during expiration and the minimum diameter during inspiration (IVCmin). The IVC collapsibility index (IVC-CI) is calculated using the standard formula [(IVCmax − IVCmin)/IVCmax] × 100. The IVC-CI inversely correlates with the central venous pressure. The negative thoracic pressure in inspiration increases the caval blood flow and decreases the intraluminal pressure. The venous return to the right atrium increases, accompanied by IVC emptying, and the diameter of the IVC decreases. (**c**) Hypovolemia: complete collapsibility of the IVC during inspiration. (**d**) Overhydration: insufficient collapsibility of the IVC during inspiration.

Lung ultrasound scanning (LUS). LUS was performed at the bedside using a commercially available device (My Lab 7, Esaote SpA, Genoa, Italy) equipped with a linear probe (frequency: 7–12 MHz). B-lines, defined as hyperechogenic vertical reverberation artifacts arising from the pleural line, were quantified in 14 intercostal positions on the anterior and lateral chest wall, from the 2nd to the 5th intercostal space on the right hemithorax and from the 2nd to the 4th intercostal space on the left hemithorax (Figure 1) [17]. We recorded the maximum number of B-lines visualized at each site of the chest. In each position, B-lines were quantified from 0 to 10, and the final score was calculated as the sum of the 14 regional scores. We chose a 14-position method to quantify the number of B-lines because it showed a good correlation with the degree of hypervolemia in previous studies [17,20,21,22]. According to the literature, a value of <5 B-lines was considered indicative of euvolemia and/or hypovolemia [17].

Inferior vena cava (IVC) scanning. The evaluation of the IVC diameter by ultrasound during spontaneous breathing is a recognized tool to assess intravascular volume and preload [23]. The IVC was explored in the subxiphoid window in its sagittal view with a curvilinear probe (2–6 MhZ). After visualization of the IVC, a loop was acquired, maximizing the IVC diameter throughout the respiratory cycle. The maximum IVC diameter (IVCmax) was calculated during expiration, and the minimum diameter during inspiration (IVCmin), as previously described, and the IVC collapsibility index (IVC-CI) was calculated using the standard formula [(IVCmax − IVCmin)/IVCmax] × 100 [20]. Since the IVC-CI is not dependent on physical attributes, age, and gender, it may serve as an objective tool for monitoring the fluid status of patients [24].

LUS and IVC assessment were performed at ED admission at our tertiary hospital to assess the volume status and were correlated with the variation in the patient’s weight (expressed as a percentage). Both LUS and IVC were performed by expert sonologists, with several years of previous experience.

### 2.5. Controls

Quantification of B-lines was performed in a control group of children with ESKD treated with haemodialysis and peritoneal dialysis, and who experienced episodes of symptomatic fluid overload between 1 June 2018 and 1 November 2020, in order to obtain a B-line threshold indicative of severe/symptomatic lung congestion. The same assessment was performed on a cohort of healthy children admitted to the ED from 1 June 2018 to 1 November 2020, for minor injury and without renal or lung-related diseases and without clinical signs of fluid overload.

### 2.6. Outcomes

Dialysis need, neurological involvement (defined as encephalopathy, altered mental status, focal neurological deficit, seizures, and/or coma) and/or death during the acute phase were adopted as the primary composite outcome, since they reflect the severity of the disease and can have a significant impact on patient outcomes, as already described in children with STEC-HUS [6,8,12]. As a further kidney outcome, the presence or absence of CKD at 6-month follow-up was recorded. CKD was defined as stage G1–G5, according to the KDIGO guidelines [25]. The creatinine-based Schwartz formula was used to calculate the estimated glomerular filtration rate (eGFR).

### 2.7. Statistical Analysis

Parametric data were reported as the mean ± standard deviation (SD), and nonparametric data as the median and 25th–75th interquartile range (IQR). Continuous variables were compared using a non-parametric Mann–Whitney test, while proportions were compared using a Chi-square test or Fisher’s exact test. A two-tailed *p*-value of <0.05 was set as an indicator of statistical significance. Statistical analysis was performed using the SPSS 22.0 software package (IBM, Armonk, NY, USA).

## 3. Results

### 3.1. Lung Ultrasound Can Distinguish Euvolemia and Lung Congestion in Children without STEC-HUS

**Figure 2:**
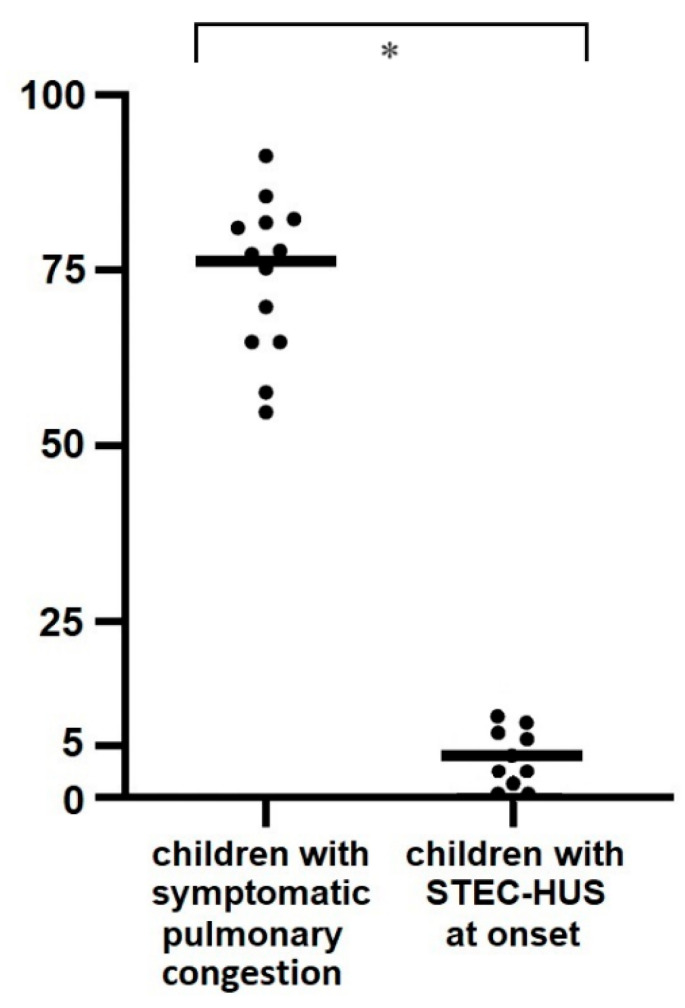
Number of B-lines in children with STEC-HUS at onset and in children on chronic dialysis therapy with severe symptomatic lung congestion. Considering as safe a maximal cut-off of 30 B-lines (suggestive of mild-to-moderate lung congestion), the quantification of the B-lines would potentially allow for maximizing the rehydration of children with HUS, minimizing the risk of pulmonary oedema. The asterisk represents a *p*-value < 0.001.

To set-up the use of LUS in identifying euvolemia and lung congestion in children (i.e., the number of B-lines corresponding to either condition), we first performed LUS in 53 children admitted to the ED not showing kidney and lung disease (healthy controls). The median age at the LUS assessment was 3.8 years (range 0.1–15.4 years). LUS showed a median value of 2 B-lines (range 0–7), corresponding to euvolemia or mild hypovolemia. Additionally, we performed LUS in a group of 13 children on chronic dialysis due to diseases other than STEC-HUS who experienced symptomatic lung-congestion episodes. Quantification of B-lines during the acute episodes showed an average of 76 B-lines (range 58–96) (Figure 2), a value indicative of severe lung congestion. Taken together, these results suggest that LUS can identify symptomatic lung congestion by a high number of B-lines (>30 B-lines) and euvolemia or hypovolemia by the absence of or few B-lines (<5 B-lines), as they are significantly different (*p* < 0.001).

### 3.2. Hypovolemia Is Frequent in Children with STEC-HUS, Irrespective of Massive Volume Administration

After applying inclusion and exclusion criteria, a total of 10 children with STEC-HUS were enrolled in the study (Table 1). The mean age at disease onset was 3.9 (range 1.4–9.5 years). All patients showed gastrointestinal symptoms (bloody diarrhoea and/or vomiting) at ED assessment, with a median symptom duration of 5.5 days (range 3.0–7.0 days). The median duration of hospitalization was 12.0 days (range 7.0–34.0 days). At the time of ED admission, although euvolemic, signs of clinical fluid overload were identified in 6 children by the presence of periorbital oedema (3/9, 33.3%), feet oedema (1/9, 11.1%) and hypertension (3/9, 33.3%; Table 1). Two patients showed clinical signs of fluid overload, even if significantly dehydrated. All patients showed mild-to-moderate hypoalbuminemia (median: 2.4 g/dL, range 1.6–3.3 g/dL). Three patients showed neurological complications during the clinical course: two experienced seizures, and one was in a coma at ED admission. Oligoanuria was found in 8/10 (80%) patients, all requiring kidney replacement therapy by haemodialysis. Among them, after six months, three patients recovered normal kidney function, one developed persistent proteinuria, two developed CKD stage 2, and one patient had CKD stage 4.

According to the extent of hydration therapy before ED admission, patients were classified into EV+ (*n* = 6) and EV− (*n* = 4).

### 3.3. The Role of Lung Ultrasound and IVC Collapsibility Index in Children with STEC-HUS

LUS assessment at ED admission found a median of 3 B-lines (range 0–10) in patients with STEC-HUS. These values are comparable to those of controls with euvolemia or mild hypovolemia (*p* = 0.92) and significantly lower than those found in children on chronic dialysis and with symptomatic lung congestion (*p* = 0.001). The quantification of the IVC diameter and IVC collapsibility index did not show a linear correlation with the patient’s volume status, either when expressed as the number of B-lines (rs = −0.07, *p* = 0.84) or as a percentage of increase or decrease from body weight at baseline (rs = −0.25, *p* = 0.50) or when compared with clinical examination (rs = −0.42, *p* = 0.26).

Patients who experienced neurological complications were characterized by negative delta weight (average −0.3 kg), lower albuminemia (range 1.6–1.8 g/dL), B-lines < 5, IVC collapsibility index >60%, an absence of hypertension at onset (but with later appearance during hydration), and they were all in the EV− group; all these aspects suggest an underlying significant dehydration.

**Figure 3:**
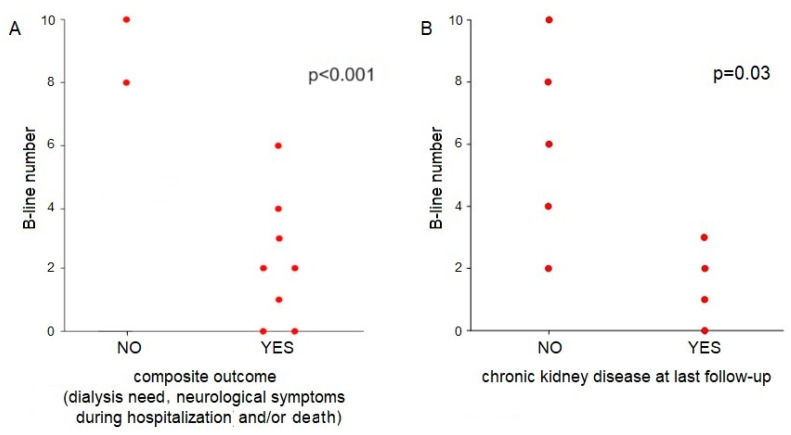
Number of B-lines at emergency department admission in relation to different outcomes. (**A**) Number of B-lines at emergency department admission in STEC-HUS children without dialysis need and without neurological impairment and VS patients with dialysis need and/or neurological involvement in the acute phase. (**B**) Number of B-lines at emergency department admission in STEC-HUS children developing or not developing chronic kidney disease at last follow-up.

We then evaluated the role of LUS performed at ED admission in predicting outcomes in patients with STEC-HUS. Patients needing dialysis and/or experiencing neurological involvement during the acute phase showed a significantly lower number of B-lines at ED assessment compared to patients with a good clinical course (2.2 vs. 9 B-lines, *p* < 0.001) (Figure 3A).

In addition, patients with normal kidney function at 6-month follow-up showed a significantly higher number of B-lines at ED admission in comparison to patients who developed CKD within the first 6 months of follow-up (6 vs. 1.5 B-lines, *p* = 0.03) (Figure 3B).

Taken together, these results suggest that a lower number of B-lines at ED admission (<5 B-lines), as an expression of lower fluid status, is associated with worse acute and long-term outcomes, including neurological complications, and increased risk of RRT and CKD.

## 4. Discussion

Assessing fluid status in children with STEC-HUS is critical in informing clinical management and outcomes. However, it still represents an unmet medical need, due to limited available tools. This pilot study shows that LUS can help clinicians assess fluid status at hospital admission, supporting clinical decisions. In particular, we found the following: (1) few patients with STEC-HUS showed clinical signs of fluid overload even if significantly hypovolemic, suggesting that physical examination has important limitations in fluid status assessment; (2) patients with neurological involvement during the acute phase and those requiring dialysis showed a significantly lower number of B-lines at admission compared to patients with a good clinical course; and (3) patients with subsequent renal impairment also presented a significantly lower number of B-lines at disease onset.

The results of this pilot study lay the foundations for a clinical trial testing the usefulness of LUS in guiding early volume-expansion therapy in these critically ill patients. Early indices of hypovolemia and poor vascular refill (hyponatremia ≤ 128 mEq/L, hypoalbuminemia, high haematocrit or haemoglobin, the number of days since the beginning of diarrhoea to ED admission, and low bicarbonate concentration on admission) previously showed a strong correlation with more severe disease in short-term and long-term prognosis, i.e., kidney failure, neurological involvement, and death [5,7,8,9]. However, these potentially prognostic parameters have several shortcomings and lack specificity in predicting hypovolemia. Consequently, there is an urgent need to explore the techniques that could help clinicians accurately measure the fluid status of patients with STEC-HUS. However, emerging as a cornerstone therapy for patients with STEC-HUS [12], early volume expansion requires an exact estimate of fluids to be administered to avoid either insufficient treatment or cardiovascular complications related to fluid overload. The need to carefully assess the volume status of the nephrological patient is well recognized in the literature, and LUS could be very useful in this sense [26]. Several studies observed correlations between LUS measurements and clinical volemic parameters, such as the residual weight after dialysis and the interdialytic weight gain [27,28]. The LUST study showed that lung congestion identified by physical examination (through crackles and/or oedema) poorly reflects the severity of congestion as detected by the number of B-lines at LUS in patients with ESKD [29]. A pilot study on 39 adult patients with AKI confirmed that LUS has the potential to detect lung congestion at a pre-clinical state (48% of patients had lung congestion detected by LUS vs. only 32% who were clinically hypervolemic) [17].

In our study, patients with dialysis need and/or neurological involvement in the acute phase of the disease showed a significantly lower number of B-lines at ED admission as an expression of a worse degree of hypovolemia. Furthermore, we observed a significantly lower number of B-lines at ED admission in children who developed CKD at the 6-month follow-up. Interestingly, none of these patients followed the early volume-expansion protocol. These data are in agreement with the study conducted by Ardissino et al. [6], reporting that hypovolemia is related to worse nephrological and neurological outcomes in patients with STEC-HUS. Furthermore, Oualha et al. [30] showed that in a cohort of 49 children with STEC-HUS, the duration of dialysis greater than 10 days was significantly associated with hypovolemia at onset. It therefore seems clear that the worse the hypovolemia (and therefore, the lower the number of B lines), the worse the outcome. Interestingly, there was no correlation in our patients between the number of B-lines and the state of hydration on physical examination, thus highlighting the fact that the number of B-lines would seem to correlate better with the prognosis of the patients than the clinical parameters. Furthermore, all our patients had a low albumin plasma level, a well-known parameter of AKI-related worst outcome occurrence [31]. As previously published, we confirmed that the presence of fewer than 5–7 B-lines corresponds to euvolemia or hypovolemia, and more than 30 B-lines corresponds to severe fluid overload [32]. From the analysis of the results of the control group, we could speculate that a low number of B-lines during intravenous infusion therapy could be used as a safety parameter to help the clinician in the maximization of volume expansion therapy without the risk of pulmonary congestion.

LUS is a radiation-free, easy-to-perform on unstable patients, and inexpensive technique. Furthermore, it is acceptable and well-tolerated by paediatric patients and their families. Despite the growing use of LUS in paediatrics [32], few studies explored its role in children with kidney diseases. Our study confirmed the efficiency of LUS as a practical and sensitive method of quantifying subclinical fluid overload in infants and children on chronic dialysis [17]. To the best of our knowledge, this is the first study reporting the potential role of LUS in children with STEC- HUS.

This study has several limitations. First, a low sample size limits the generalizability of the results. Secondly, the estimate of dry weight, based on clinical anamnestic data or on the growth percentile of the last year followed by the child, might be inaccurate. On the other hand, it represents a pragmatic approach, especially in emergency situations such as STEC-HUS. In fact, the patient may have lost or gained weight during the last year due to multiple causes (diet, concomitant illnesses, and puberty). Thirdly, the assessment of B-lines was not performed at different time points during the hospitalization, which would have provided further information on the treatment-related change in the number of B-lines. Lastly, some patients have been transferred from other hospitals, with probable data loss and a heterogeneous application of intravenous hydration protocols.

This pilot study might be a first step, and prospective studies are needed in order to verify the efficacy and the safety of fluid administration driven by the B-line number in children with STEC-HUS in reducing long-term complications.

Fewer B-lines indicate hypovolemia/less congestion at the time of admission, but do not necessarily translate directly into worse outcomes. In clinical practice, B-line interpretation must be integrated with other clinical findings and diagnostic tests [32].

## 5. Conclusions

LUS is a practical and sensitive method for quantifying the hydration status of paediatric patients with STEC-HUS and can be a valuable aid for monitoring hydration status during fluid therapy. LUS might enable the optimization (and maximization) of hydration therapy without incurring complications from fluid overload, and prevents kidney and systemic complications related to hypovolemia and effective blood volume reduction.

## Figures and Tables

**Table 1:** Characteristics of patients, serum laboratory parameters at ED admission, and complications.

	All Patients (*n* = 10)	Clinically Hypovolemic Patients (*n* = 4)	Clinically Euvolemic Patients (*n* = 6)
**Characteristics at ED admission**			
Age (years)	3.9 (1.4–9.5)	5.5 (3.2–9.5)	2.9 (1.4–5.5)
Number of B-lines	3.6 (0–10)	5.3 (1–10)	2.5 (0–8)
IVCD max (mm)	6.5 (3–11.2)	9 (5.5–11.2)	7.4 (3–10.8)
IVCD min (mm)	4.5 (0.5–7.5)	4.7 (1.5–7.3)	4.5 (0.5–7.5)
IVC-CI (%)	49 (25–83)	50 (35–73)	48 (25–83)
EV+	6 (60)	2 (50)	4 (66)
Hypertension	4 (40)	0	4 (66)
Oligoanuria	8 (80)	4 (100)	4 (66)
**Serum laboratory parameters at onset**			
creatinine (mg/dL)	3.5 (0.7–11.4)	4.9 (0.8–11.4)	2.6 (0.7–3.6)
urea/creatinine ratio	73.6 (28–122)	68 (47–120)	78 (28–122)
Na (mEq/L)	134 (126–142)	131 (126–138)	135 (131–142)
Albumin (g/dL)	2.4 (1.6–3.3)	2.3 (1.6–3.1)	2.5 (1.8–3.3)
**Complications**			
Dialysis need during hospitalization	8 (80)	3 (75)	5 (83)
Death during hospitalization	1 (10)	0	1 (17)
Neurological involvement during hospitalization	3 (33)	2 (50)	1 (17)
CKD at last follow-up	4/9 (44)	3/4 (75)	1/5 (20)

CKD, chronic kidney disease; ED, Emergency Department; EV+, patients receiving important hydration; IVC-CI, inferior vein cava collapsibility index; IVCD, inferior vein cava diameter. Baseline data recorded at time of entry to the study, nephrological and neurologic outcomes during hospitalization. Data are presented as mean (range) or number (%).

## Data Availability

The data underlying this article will be shared on reasonable request made to the corresponding author.
